# Analysis of Galangin and Its In Vitro/In Vivo Metabolites via Ultra-High-Performance Liquid Chromatography/Quadrupole Time-of-Flight Mass Spectrometry

**DOI:** 10.3390/metabo12111032

**Published:** 2022-10-28

**Authors:** Feng Zhao, Yinling Ma, Jintuo Yin, Ying Li, Yanli Cao, Lantong Zhang

**Affiliations:** 1 School of Pharmacy, Hebei Medical University, Shijiazhuang 050017, China; 2 National Clinical Drug Monitoring Center, Department of Pharmacy, Hebei Province General Center, Shijiazhuang 050051, China

**Keywords:** galangin, UHPLC-Q-TOF-MS/MS, in vitro and in vivo, metabolic profile, multiple mass defect filter

## Abstract

Galangin, a naturally available flavonoid, induces a variety of pharmacological activities and biological effects via several mechanisms. However, in vivo metabolism of galangin has not been fully explored, which means knowledge of its pharmacodynamics and application potential is limited. The objective of this study was to establish an ultra-high-performance liquid chromatography–quadrupole time-of-flight mass spectrometry method for the rapid profiling and identification of galangin metabolites in vitro and in vivo using unique online information-dependent acquisition with multiple mass defect filtering combined with dynamic background subtraction in positive ion mode. A total of 27 metabolites were detected and characterized, among which eight metabolites in liver microsomes and four metabolites in intestinal microflora were characterized, and 27 metabolites from rat plasma, bile, urine, feces, and a number of different tissue samples were characterized. Thirteen major metabolic pathways including hydrogenation, hydroxylation, glycosylation, methylation, acetylation, glucuronidation, and sulfation were observed to be attributable to the biotransformation of the metabolites. This study provides evidence for the presence of in vitro and in vivo metabolites and the pharmacokinetic mechanism of galangin. Moreover, the study promotes the further development and utilization of galangin and the plant from which it is derived, *Alpinia officinarum* Hance.

## 1. Introduction

**Figure 1:**
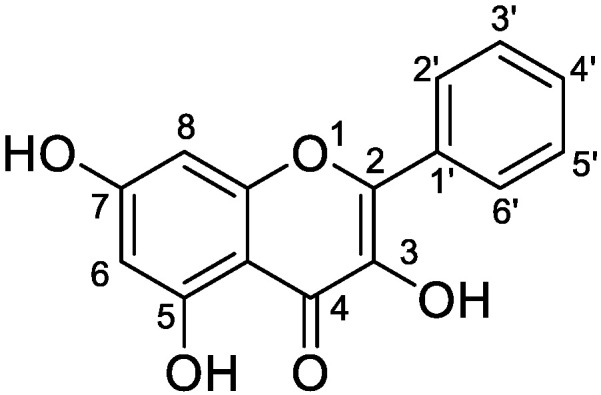
Chemical structure of galangin.

Galangin (3,5,7-trihydroxyflavone; Figure 1), a natural flavonol compound isolated from the root of Alpinia officinarum or lesser galangal, a rhizome plant from the Zingiberaceae family, is emerging as a promising anticancer agent, exerting anticancer effects via several mechanisms, having both chemopreventive and therapeutic effects against different types of cancer [1,2,3,4,5,6,7,8]. Besides, galangin could attenuate diabetic cardiomyopathy through modulating oxidative stress, inflammation, and apoptosis [9]. In addition, it could manage melanin biosynthesis through inhibition of tyrosinase [10]. Recently, galangin was reported to be a potential antithrombotic drug candidate [11]. In recent years, reports on galangin have focused on the pharmacokinetics in rats and its effects on CYP450 enzymes [12,13,14]. These reports provide a theoretical basis for studies on the galangin delivery process in vivo and drug interactions. However, to date, there are no reports on the galangin metabolic profile. As metabolites can significantly influence the efficacy and safety of drugs and may have different biological properties from the parent drug, the elucidation of the structure of galangin metabolites is essential for understanding the efficacy and safety of galangin.

In recent years, with the development of data acquisition and analysis technology, high-resolution mass spectrometry has been widely used for metabolite detection owing to its rapid turn-around times and high sensitivity [15,16]. In addition, information-dependent acquisition (IDA) has been combined with multiple data mining techniques such as dynamic background subtraction (DBS) and multiple mass defect filtering (MMDF) to trigger MS/MS data acquisition for all possible metabolites, especially in complex biological matrices, where metabolites are acquired at much lower concentrations in a single injection cycle [17]. In addition, several offline data processing techniques, such as extraction ion chromatography, mass defect filtering, product ion filtering (PIF), and neutral loss fragment (NLF) studies, have been successfully applied to metabolite identification.

Herein, a highly sensitive and specific ultra-high-performance liquid chromatography–quadrupole time-of-flight mass spectrometry (UHPLC-Q-TOF-MS/MS) assay with multiple data mining methods was established to identify galangin and its metabolites in vitro and in vivo. Moreover, the potential metabolic pathways of galangin were proposed in this study. To the best of our knowledge, this is the first study to comprehensively investigate the in vitro (rat liver microsomes and intestinal bacteria) and in vivo (rat plasma, bile, urine, feces, and a variety of tissue samples) metabolisms of galangin. This study provides essential information for understanding the metabolism and excretive pharmacokinetics of galangin in humans and rats.

## 2. Materials and Methods 

### 2.1. Chemicals and Reagents

Galangin (98.3% purity) was obtained from Nanjing Plant Origin Biological Technology (Nanjing, China). As reference standards, chrysin (98.5% purity), kaempferide (98.7% purity), apigenin (98.5% purity), and kaempferol (98.0% purity) were supplied by the National Institute for the Control of Pharmaceutical and Biological Products (Beijing, China). Rat liver microsomes (RLMs) were laboratory-made at the Department of Pharmaceutical Analysis in School of Pharmacy of Hebei Medical University and stored at −80 °C until used. Phosphate-buffered saline (PBS), β-Nicotinamide adenine dinucleotide phosphate (NADPH), Uridine 5′-diphosphoglucuronic acid (UDPGA), and alamethicin were provided by Solarbio Science Co., Ltd. (Beijing, China). Magnesium chloride hexa-hydrate, sodium carboxymethyl cellulose (CMC-Na), and other chemicals were of analytical grade (Tianjin Chemical Corporation, Tianjin, China). Biological saline was obtained from Shijiazhuang No. 4 Pharmaceutical (Shijiazhuang No. 4 Pharmaceutical Co., Ltd., Shijiazhuang, China). Methanol and acetonitrile (HPLC grade) were purchased from Merck Company (Darmstadt, Germany); formic acid (LC/MS grade) was purchased from Fisher Scientific (Fairlawn, NJ, USA); they were utilized to prepare stock or working solutions, the mobile phases. Ultra-pure water was acquired from Wahaha Group Co., Ltd. (Hangzhou, China). All other chemicals and reagents were analytical grade and are commercially available.

### 2.2. In Vivo Metabolism

#### 2.2.1. Animal and Drug Administration

Male Sprague–Dawley rats (200–220 g body weight, 6–8 weeks old) were obtained from the Laboratory Animal Centre of Hebei Medical University (Certificate No. SCXK (Ji) 2018-003, Shijiazhuang, China). The rats were housed in controlled environmental conditions (22–25 °C, 50–60% relative humidity, 12:12 h light/dark cycle) and fasted for 12 h before administration of galangin with free access to water. The animal experiments were conducted according to the guidelines for animal experimentation at Hebei Medical University, and the animal protocols were approved by the Animal Ethics Committee of Hebei Medical University.

The 24 rats were randomly divided into four groups, from which plasma, urine and feces, bile, and organs and tissue were collected, respectively. Each group was divided into two groups: a control group (CON) and galangin-treated group (TRE). The rats in the experiment groups were administered galangin by gavage; galangin was dissolved in 0.5% carboxymethyl cellulose sodium (CMC-Na) solution at 100 mg/kg. The rats in the control groups were orally administered the equivalent volume of CMC-Na solution with no galangin.

#### 2.2.2. Sample Collection of Plasma, Urine, Feces, Bile, and Tissue

Plasma sample collection: After oral administration, plasma samples (0.5 mL) were obtained from the suborbital venous plexus of the rats in the TRE at 0.083, 0.17, 0.5, 1, 2, 4, 6, 8, 12, and 24 h. The plasma samples were transferred to micro-tubes containing 5 μL of heparin and then immediately centrifuged for 15 min (1500× *g*, 4 °C), and the plasma supernatant from every sample in the TRE was combined to obtain a composite plasma sample containing the drug. The blank plasma sample was from the CON, and the method of collection was the same as for the test samples. The above-mentioned plasma samples were separated and immediately frozen at −80 °C until required for further analysis.

Urine and feces collection: Urine and fecal samples were collected at 0–4 h, 4–8 h, 8–2 h, 12–24 h, 24–36 h, 36–48 h, 48–60 h, and 60–72 h after administration using separate metabolic cages (type DXL-DL, Suzhou Fengshi Laboratory Animal Equipment Co., Ltd., Suzhou, China). The urine and fecal samples were immediately centrifuged for 15 min (3500 rpm, 4 °C) and were combined in the TRE to obtain a composite sample containing the drug. The blank urine was from the control group, and the method of collection was the same as for the test samples.

Bile collection: The rats were injected with urethane physiological saline solution (1.5−2 g/kg) after gavage and bile duct cannulation. Then, the bile samples were collected at 0−1 h, 1−3 h, 3−5 h, 5−8 h, 8−12 h, 12−20 h, and 20−24 h after oral administration. The test sample was centrifuged and combined according to the same method as specified for the urine sample.

Organ and tissue collection: At 4 h and 12 h post-dosing, the organs and tissue (including the heart, liver, spleen, lung, kidney, stomach, muscle, brain, intestine, testicles, and medullary) were harvested and rinsed with ice-cold 0.9% saline to remove the superficial plasma. After being blotted dry with filter paper, each weighed tissue sample was homogenized using a tissue homogenizer in physiological saline solution (1:2, *w*/*v*).

#### 2.2.3. Preparation of Plasma, Urine, Feces, Bile, and Tissue Sample Solutions

Acetonitrile was added to the amalgamated plasma, urine, feces, bile, and tissue samples (3-fold dilution) and to 2.0 g of feces, and the samples were extracted by ultrasound for 45 min. Then, the supernatants were concentrated to dryness under a nitrogen atmosphere at 25 °C. Blow-dried samples were dissolved in 600 μL acetonitrile and vortex mixed for 5 min. After the mixture was centrifuged at 10,000× *g* for 10 min, the supernatant (3 μL) was injected into the UHPLC-Q-TOF-MS/MS system for analysis. All biosamples were stored in a −80 °C refrigerator.

### 2.3. In Vitro Metabolism by Rat Liver Microsomes

Rat liver microsomes were prepared according to a previously described method [18]. Ten male rats were anesthetized with ether and fixed on a wooden plate. The liver was removed from the abdominal incision, perfused, and washed with ice-cold potassium phosphate buffer (10 mM, pH 7.4). The fresh rat livers were harvested, rinsed, and weighed. The livers were minced and homogenized using a motorized homogenizer in ice-cold potassium phosphate buffer and centrifuged at 5000× *g* for 15 min at 4 °C. The supernatant was mixed and centrifuged again at 12,000× *g* for 15 min at 4 °C. The precipitate was discarded, and the supernatant was then centrifuged at 100,000× *g* for 1 h at 4 °C to yield the microsomes. The total concentration of microsomal protein was determined using the Bio-Rad protein assay Kit (Bio-Rad laboratories, Hercules, CA, USA) with bovine serum albumin as a standard. The qualified liver microsomes were distributed and stored at 80 °C until use.

Phase I metabolism of galangin was conducted by liver microsomes (1.0 mg protein/mL), 3.3 mmol/L MgCl_2_, 1.3 mmol/L β-NADPH, 0.1 mol/L phosphate buffer (PBS, pH 7.4), and 100 μmol/L galangin (total volume = 200 μL). The incubation mixture was pre-incubated at 37 °C for 10 min. Metabolism was initiated by adding NADPH, and the mixture was further incubated at 37 °C. After incubation at 37 °C for 1.5 h, the reaction was terminated by adding 200 μL of ice-cold acetonitrile. The samples were vortexed for 3 min and centrifuged at 10,000× *g* for 10 min, and 3 μL of supernatant was injected into the UHPLC-Q-TOF-MS/MS system for analysis. The control sample was incubated without NADPH following the same treatment, and the blank sample was incubated with or without galangin.

The phase II metabolism reaction was performed with liver microsomes (1.0 mg protein/mL), 3.3 mmol/L MgCl_2_, 2.0 mmol/L UDPGA, 25 μg/mL alamethicin in PBS (pH 7.4), and 100 μmol/L galangin. The reaction was initiated via adding the UDPGA-generating system, and the incubation procedure and sample pretreatment were identical to those for the above-mentioned phase I metabolic reaction. The control sample was incubated without UDPGA and followed the same treatment, and the blank sample was incubated with or without galangin.

### 2.4. In Vitro Metabolism by Rat Intestinal Flora

The general anaerobic medium broth [18,19] was prepared as follows: KH_2_PO_4_ (37.5 mL, 0.78% (*w*/*v*)), Solution A (37.5 mL, 0.47% (*w*/*v*) KH_2_PO_4_, 1.18% NaCl, 1.2% (*w*/*v*) (NH_4_)_2_SO_4_, 0.12% (*w*/*v*) CaCl_2_, and 0.25% (*w*/*v*) MgSO_4_), Na_2_CO_3_ (50 mL, 8% (*w*/*v*)), L-cysteine (0.5 g), L-ascorbic acid (2 mL, 25% (*w*/*v*)), eurythrol (1 g), tryptone (1 g), and nutrient agar (1 g) were weighed and dissolved in 1000 mL of distilled water. Then, the pH was adjusted to 7.5–8.0 at 25 °C with 1 M HCl. The anaerobic culture medium was autoclaved at 121 °C for 20 min and used immediately.

Fresh fecal samples were obtained from healthy male rats, mixed, and immediately homogenized. Fresh fecal samples (1 g) were weighed and suspended in 4 mL of sterile physiological saline in a centrifuge tube and vigorously stirred and filtered with medical gauze to obtain an intestinal flora culture solution. Galangin dissolved in 200 μL of methanol (1 mg/mL) was incubated at 37 °C for 24 h in an anaerobic culture bag (AnaeroPack, Mitsubishi Gas Chemical Co., Inc., Tokyo, Japan), which was filled with sufficient nitrogen. After incubation for 24 h at 37 °C, the reaction was terminated by adding 3 volumes of acetonitrile. The control sample was incubated without intestinal flora culture solution following the same treatment, and the blank sample was incubated without galangin. The sample pretreatment was the same as that for the urine sample.

### 2.5. Instruments and Analytical Conditions

The high-mass-resolution UHPLC-Q-TOF-MS/MS system was used for metabolite identification. It consisted of a Shimadzu LC-30A UHPLC system (Shimadzu, Kyoto, Japan), equipped with a binary solvent delivery system, an online degasser, an autosampler, a column compartment, and a quadrupole time-of-flight TripleTOF™ 5600+ mass spectrometer equipped with positive ESI mode. Chromatographic separation was performed on a 2.7 μm Poroshell 120 EC-C_18_ reversed-phase column (2.1 mm × 100 mm, Agilent Technologies, Little Fall, DE, USA) with a security guard UHPLC C_18_ column at 40 °C. The mobile phase consisted of Eluent A (0.1% aqueous formic acid) and Eluent B (acetonitrile) delivered at a flow rate of 0.3 mL/min using the following gradient program: 0–2 min, 10–15% B; 2–12 min, 15–60% B; 12–20 min, 60–95% B. The run time was 20 min, and the post-run time was 5 min. The autosampler was maintained at 4 °C. The injection volume was 3 μL for each sample and reference compound solution. For the full-scan acquisition mode at *m/z* 100−1000 Da, the TOF parameters were as follows: ion spray voltage, 5.5 Kv; ion source heater, 550 °C; DP, 60 V; CE, 35 eV; CES, 15 eV; drying gas, N_2_; flow rate, 10 L/min; curtain gas, 35 psi; nebulizer gas, 55 psi; heater gas, 55 psi. The IDA method consists of a TOF-MS measurement scan (*m/z* 100–800), followed by eight TOF-MS/MS scans (*m/z* 50–800) with accumulation times of 0.25 s and 0.1 s, respectively. In addition, the 12 most-intense fragment ion signals for each metabolite were selected by product ion scanning only when they exceeded 50 cps counts, and the accumulation time was 200 ms in both positive and negative ion modes. In addition, an automated calibration delivery system was used to regulate the MS and MS/MS automatically.

### 2.6. Analytical Strategy

The analysis was performed in three steps: First, the biological samples were injected into the UHPLC-Q-TOF-MS/MS, and online data acquisition was performed using the Analyst™ software 1.6.2; accurate MS/MS datasets were obtained using the MMDF and DBS-dependent data acquisition techniques. Moreover, MMDF templates were established according to literature reports and the pre-experiment results. For the subsequent post-acquisition data mining processing, multiple data mining methods including high-resolution extracted ion chromatogram, MMDF, NLF, and PIF were utilized to investigate the galangin metabolites. Among them, the analyses of the prototypes and metabolites were performed with MetabolitePilot™ 1.5 (AB SCIEX, Framingham, MA, USA) based on accurate measurements of the *m/z* values. Additionally, the positional isomers were distinguished according to the retention times and Clog *p*-values. Depending on the information obtained above, all metabolites were identified. Finally, with consideration of the metabolite data and references, the metabolic pathway of galangin was proposed.

## 3. Results and Discussion

### 3.1. Optimization of LC-MS Conditions

The simultaneous determination of galangin metabolites under one set of chromatographic conditions is challenging due to the differences in the polarity and content of different types of galangin metabolites. Adequate chromatographic separation is one of the most important aspects of metabolite identification, as it prevents metabolite masking. To obtain the optimal separation effects and sensitivity, different mobile phase conditions were compared, and methanol–water, which has low toxicity and a low cost, was selected as the mobile phase. The ionization effect was better when 0.1% formic acid was added to the mobile phase. These conditions can be used to separate galangin and its metabolites from endogenous substances in a biological matrix.

The metabolites of galangin have diverse structures. Some metabolites respond well to positive ions, while some metabolites produce more mass spectrum information in negative ion mode. To obtain more comprehensive mass spectrum information and elucidate the structure of the compounds, we adopted positive and negative modes of detection in preliminary experiments and optimized the fragmentation voltage (DP), collision energy (CE), and collision energy spread (CES). Considering that almost all metabolites were suitable for analysis in positive ion mode, we selected positive ion mode for detection.

### 3.2. Analytical Strategy and Metabolite Identification

**Figure 2:**
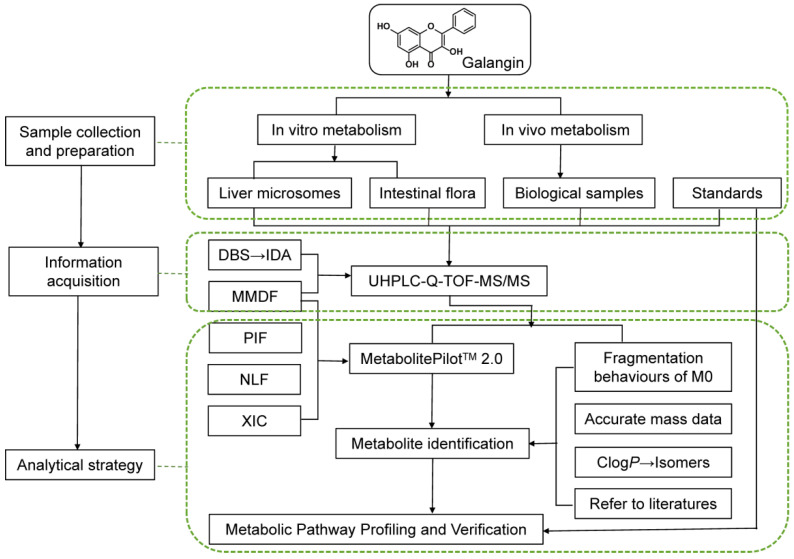
Work flow of the analytical procedure for identification of galangin metabolites.

A comprehensive and efficient strategy (Figure 2) was established for the integrated screening and characterization of the galangin metabolites using UHPLC-Q-TOF-MS/MS, combined with online data acquisition and multiple data post-processing technologies.

To avoid the influence of background noise and complex endogenous compounds, UHPLC-Q-TOF-MS/MS was used to acquire mass spectrometry data at *m/z* 100~1000 in positive ion mode, and the MMDF and DBS online data acquisition techniques were used to track all possible metabolites. Developing the MMDF and DBS data acquisition templates is a critical step, especially for low levels of non-predicted metabolites, which can reduce the potential interference from endogenous substances and trigger a second scan to obtain rich mass spectral information for structure determination. Homologues may have identical backbones and similar fragmentation behavior, which means they produce similar fragmentation and the same product ions or neutral losses. With reference to the structure of galangin and the general biotransformation mode of flavonols, five templates were established (Table 1). The mass loss of possible target compounds should be within 25 mDa, so as to eliminate interference and meet the requirements for determining metabolites in biological matrix. In addition, DBS was switched on to distinguish between background ions and drug ions. In addition, NLF analysis can be used to reduce interference from non-target compounds, enabling comparisons and structure deduction. During the identification process, the chemical structures were inferred by combining the diagnostic fragment-ion-based extension strategy, NLF, and mass spectrometry fragmentation rules, which allowed the rapid identification of galangin metabolites. Third, positional isomers were distinguished by the corresponding Clog *p*-values calculated using ChemDraw 20.0 based on the different retention times. Based on the information obtained above, all metabolites were positively or putatively identified. Finally, the metabolic pathways of galangin were proposed based on the metabolite data and literature data [20,21,22,23].

**Figure 3:**
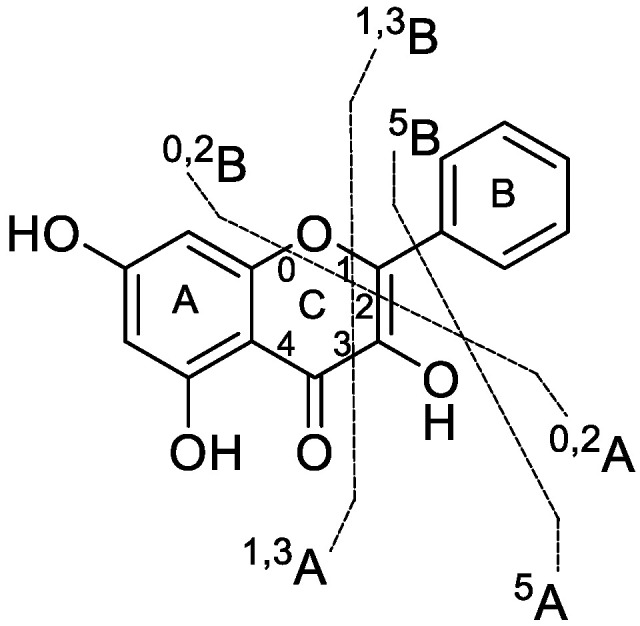
Typical cleavage pattern for galangin.

Fragmentation pattern analysis of the parent compound is of significant value for identifying the metabolites. Galangin eluted at 12.03 min with a deprotonated molecular ion [M+H]+ at *m/z* 271.0610, and the molecular formula was determined to be C_15_H_10_O_5_ according to the chromatographic behavior and MS fragmentation pattern data. The typical cleavage pattern for galangin is shown in Figure 3.

According to the MS/MS spectrum, the product ions of galangin were detected at *m/z* 242.0580, 225.0556, 215.0716, 197.0605, 165.0191, 153.0192, 119.0498, 105.0335, 91.0545, and 77.0391. The characteristic ions at *m/z* 225.0556 and 197.0605 were obtained after sequential loss of CO_2_ and CO; the typical ions at *m/z* 242.0580 and 215.0716 were generated after the successive loss of CO. Moreover, several ions at *m/z* 153.0192 (1,3A+), 119.0498 (1,3B+), 165.0191 (0,2A+), 105.0335 (0,2B+), 91.0545 ([0,2B-O]+), and 77.0391 (5B+) were present in the MS2 spectrum; the product ions at *m/z* 153.0192, 119.0498, 165.0191, and 105.0335 were formed by a retro-Diels−Alder reaction (RDA reaction) [24].

**Figure 4:**
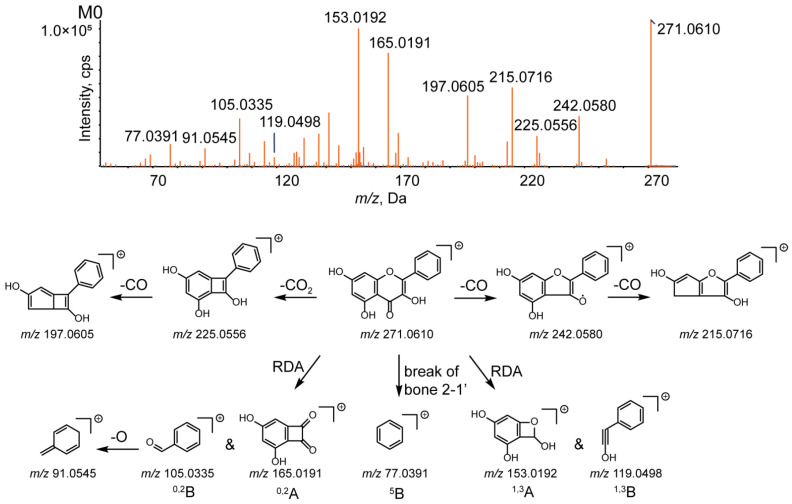
MS/MS spectrum of galangin and its proposed fragmentation pathways.

**Figure 5:**
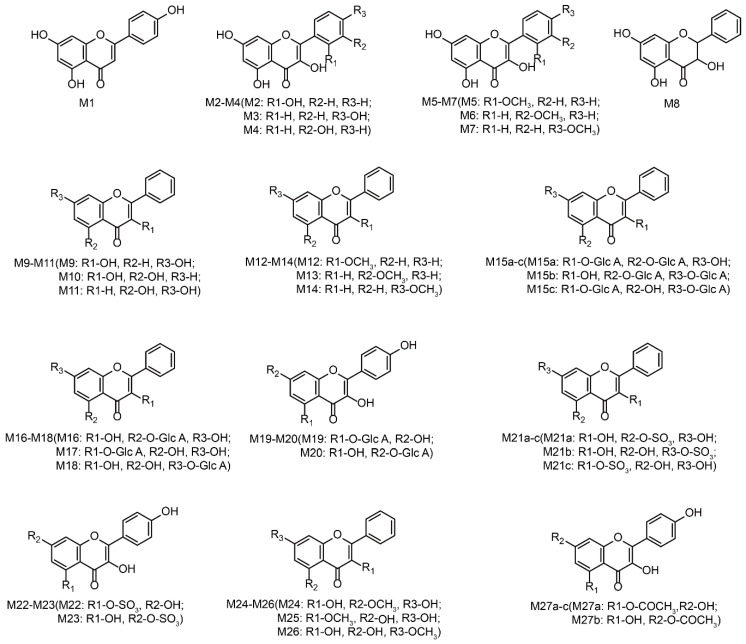
Chemical structures of all metabolites of galangin in in vitro and in vivo experiments (a, b, and c: possible chemical structure).

**Figure 6:**
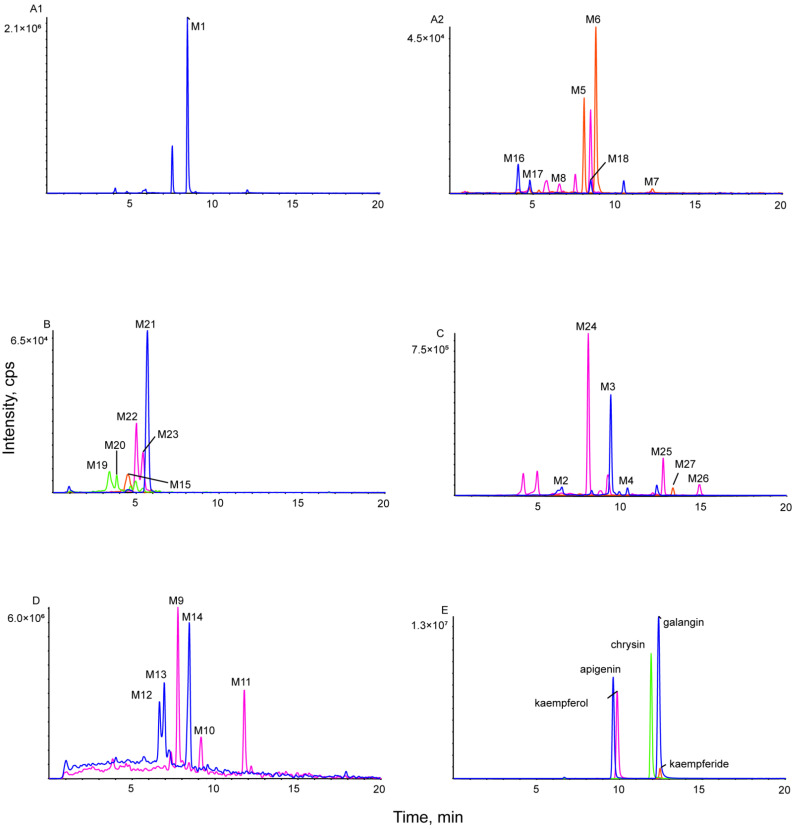
Extracted ion chromatograms of all metabolites of galangin in vitro and in vivo (**A1**,**A2**) in rat blood sample, (**B**) in rat bile sample, (**C**) in rat urine sample, (**D**) in rat fecal sample, and (**E**) in mixed standard solution.

**Figure 7:**
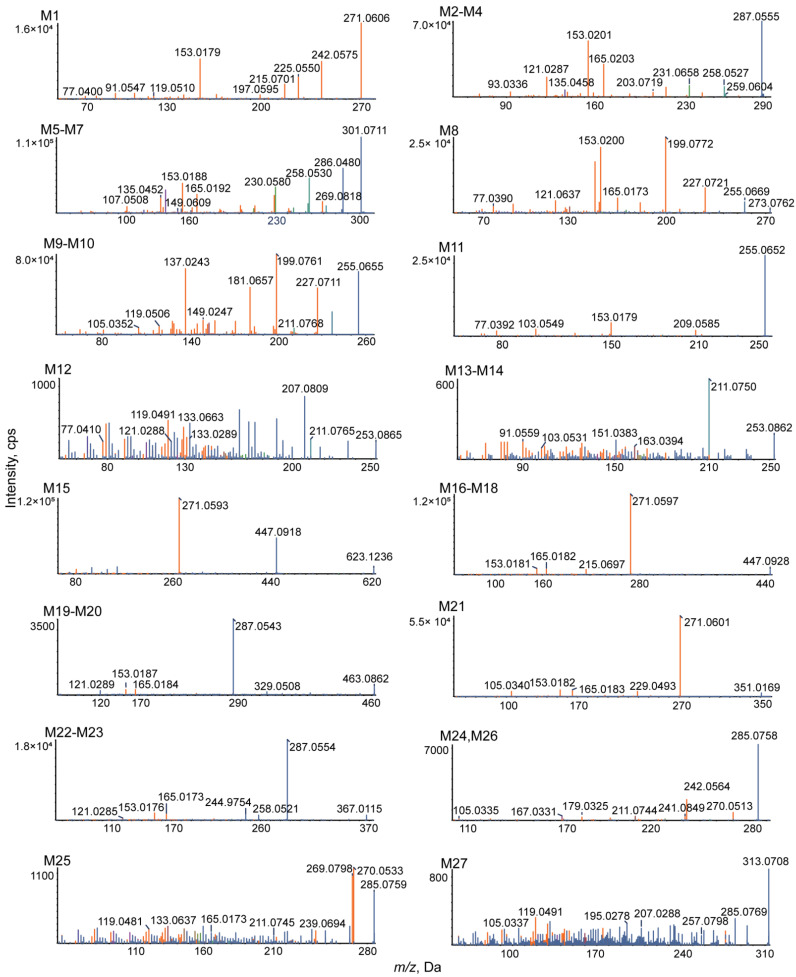
MS/MS spectra of all metabolites of galangin in vitro and in vivo.

**Figure 8:**
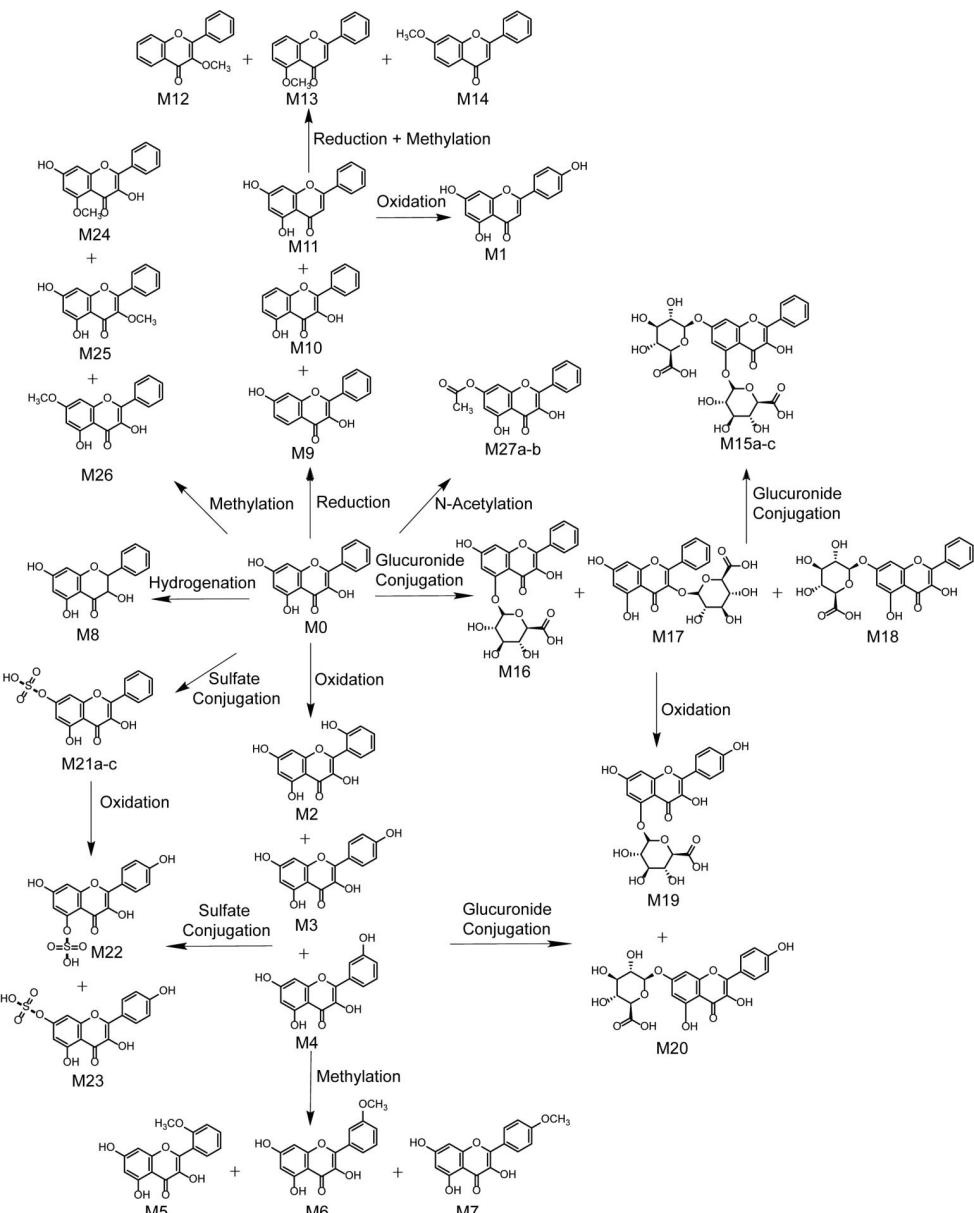
Metabolic profile and proposed metabolic pathways of galangin in vitro and in vivo (a, b, and c: possible chemical structure).

Based on the results, the MS/MS spectrum and fragmentation pathways of galangin (M0) are shown in Figure 4. All information on the metabolites of galangin is shown in Table 2. All chemical structures of the metabolites and the extracted ion chromatograms of the metabolites are shown in Figure 5 and Figure 6, respectively. The MS/MS spectra of all metabolites of galangin in vitro and in vivo are shown in Figure 7. The metabolic profile and proposed metabolic pathways are shown in Figure 8.

### 3.3. Identification of Phase I Metabolites

#### 3.3.1. Oxidation Reaction

Figure 7 shows the MS/MS spectrum of the deprotonated metabolite **M1**, which had a retention time of 8.45 min and produced a [M+H]^+^ ion signal at *m/z* 271.0606, indicating that it has the same molecular formula as the parent drug. The parent ion produced typical characteristic fragment ion signals at *m/z* 242.0575 ([M-CHO+H]^+^), 225.0550 ([M-CO_2_ +H]^+^), 215.0701 ([M-2CO+H]^+^), 197.0595 ([M-CO_2_-CO+H]^+^), 153.0179 (RDA, ^1,3^A), 119.0510 (RDA, ^1,3^B), 91.0547 ([^0,2^B-O+H]^+^), and 77.0400 ([^5^B-O+H]^+^). **M1** was found to have a molecular formula of C_15_H_10_O_5_ and a Clog *p*-value of 2.90529. **M1** was identified as apigenin through comparisons with the reference substance and literature data [20].

The quasi-molecular ions **M2** (*m/z* 287.0555), **M3** (*m/z* 287.0547), and **M4** (*m/z* 287.0556) were found to represent C_15_H_10_O_6_, based on their MS/MS results, which reveals that they are 16 Da heavier than M_0_. However, the chromatographic retention times were 6.47 min, 9.39 min, and 10.40 min, which indicates that the compounds are isomers. **M2**–**M4** produced dissociation fragment ion signals at *m/z* 259.0604, 231.0658, and 203.0719, which indicate the successive loss of CO, and a secondary fragment at *m/z* 258.0527 indicates the loss of CHO, meaning that galangin oxidation had occurred. In addition, the typical fragment ion signals at *m/z* 165.0203 (RDA, ^0,2^A), 153.0201 (RDA, ^1,3^A), 135.0458 (RDA, ^1,3^B), 121.0287 (RDA, ^0,2^B), and 93.0336 (^5^B) were formed by an RDA reaction, indicating that oxidation occurred in the B ring of the flavonoid. Furthermore, their Clog *p*-values were calculated to be 1.79989, 2.09989, and 2.09989 using ChemDraw 20.0. **M3** was identified as kaempferol by comparisons with a reference substance and literature data [21].

**M5**, **M6**, and **M7** weighed 30 Da more than M_0_ and 14 Da more than **M2**–**M4**. They produced [M+H]^+^ ions at *m/z* 301.0711, 301.0715, and 301.0712 (C_16_H_12_O_6_) with retention times of 8.08 min, 8.77 min, and 12.15 min, respectively. In their MS/MS spectrum, the fragment ion at *m/z* 286.0480 [M+H-CH_3_]^+^ was further dissociated by the loss of O or the loss of CO or 2CO, to generate *m/z* 269.0818, *m/z* 258.0530, and 230.0580, which demonstrates that galangin was oxidized and methylated; the characteristic product ions at *m/z* 165.0192 (^0,2^A), 153.0188 (^1,3^A), 149.0609 (^1,3^B), 135.0452 (^0,2^B), and 107.0508 (^5^B) were generated by RDA, indicating that both the oxidation and methylation reactions occurred in the B ring. The Clog *p*-values of **M5**–**M7** were 2.13699, 2.69699, and 2.69699, respectively. Co-chromatography with an authentic standard established that **M7** was kaempferide [22].

#### 3.3.2. Reduction Reaction

**M8** produced a deprotonated molecular ion at *m/z* 273.0762 ([M+H]^+^) with a retention time of 6.63 min. Its MS/MS spectrum revealed characteristic product ion signals at *m/z* 255.0669 ([M-H_2_O+H]^+^), 227.0721 ([M-H_2_O-CO+H]^+^), 199.0772 ([M-H_2_O-2CO+H]^+^), 165.0173 (RDA, ^0,2^A), 153.0200 (RDA, ^1,3^A), 121.0637 (RDA, ^1,3^B), and 77.0390 (^5^B). The fragment ion signal at *m/z* 121.0637 suggests that **M8** may represent the hydrogenation of galangin. The molecular formula of **M8** is C_15_H_12_O_5_, and it has a Clog *p*-value of 2.03495.

**M9**, **M10**, and **M11** afforded [M+H]^+^ ion signals at *m/z* 255.0655, 255.0654, and 255.0652 (C_15_H_10_O_4_) and had retention times of 8.08 min, 8.77 min, and 12.15 min, respectively. The signals were attributed to the loss of O because they are 16 Da heavier than M_0_. The **M9** and **M10** MS/MS spectra contain characteristic product ion signals at *m/z* 227.0711 ([M-CO+H]^+^), 211.0768 ([M-CO_2_+H]^+^), 199.0761 ([M-2CO+H]^+^), 181.0657 ([M-2CO-O+H]^+^), 149.0247 (RDA, ^0,2^A), 137.0243 (RDA, ^1,3^A), 119.0506 (RDA, ^1,3^B), and 105.0352 (RDA, ^0,2^B). The presence of fragment ion signals at *m/z* 149.0247 and 137.0243 suggests that **M9** and **M10** each lost an oxygen molecule from their A ring. Similarly, in the MS/MS spectrum of **M11**, a series of product ion signals at *m/z* 209.0585, 153.0179 (^1,3^A), 103.0549(^1,3^B), and 77.0392(^5^B) was attributable to the loss of CO_2_, the RDA reaction, and 2-1′ bond cleavage, indicating the loss of an oxygen molecule from the C ring. The Clog *p*-values increased in the following order: **M9** Clog *p*, 2.49525; **M10** Clog *p*, 3.39525; and **M11** Clog *p*, 3.56275; they were consistent with their retention times. Comparisons with the reference substance and literature data [23] led to the identification of **M11** as chrysin.

Three isomers, **M12**, **M13**, and **M14**, which weighed 18 Da less than M_0_, produced [M+H]^+^ ion signals at *m/z* 253.0865, 253.0862, and 253.0861 (C_16_H_12_O_3_) at retention times of 6.55 min, 6.90 min, and 8.43 min, respectively. The MS/MS spectrum of **M12** revealed signals at *m/z* 211.0765, 207.0809, 133.0663 (^1,3^B), 133.0289 (^0,3^A), 121.0288 (^1,3^A), 119.0491 (^0,3^B), and 77.0410 (^5^A), owing to the loss of C_2_H_2_O and CO_2_, the RDA reaction, and 2-1′ bond cleavage, which indicates that two oxygen atoms on the A ring were eliminated and the C ring underwent methylation substitution. In the **M13** and **M14** MS/MS spectra, a series of product ions at *m/z* 211.0750, 163.0394 (^0,3^A), 151.0383 (^1,3^A), 103.0531 (^1,3^B), and 91.0559 (^0,3^B) indicates the loss of C_2_H_2_O and the RDA reaction, suggesting that one oxygen atom each was eliminated from the A and C ring, and the A ring underwent a methylation substitution reaction. Furthermore, the Clog *p*-values of **M12**–**M14** were 3.089, 3.5865, and 3.5865, respectively.

### 3.4. Identification of Phase II Metabolites

The metabolic profile revealed evidence of methylation, sulfation, and glucuronide conjugation. In addition, we observed that some metabolites underwent both phase I and phase II metabolic reactions.

**M15** was detected at 4.45 min with an [M+H]^+^ ion signal at *m/z* 623.1236 (C_27_H_26_O_17_). The theoretical relative molecular mass was 352 Da greater than that of galangin, which means that **M15** is most likely the product of a di-glucuronidation reaction. The typical characteristic fragment ion signals at *m/z* 447.0918 ([M-C_6_H_8_O_6_+H]^+^) and 271.0593 ([M-2C_6_H_8_O_6_+H]^+^) confirmed the formula of **M15** to be C_27_H_26_O_17_. Hence, **M15** has three possible chemical structures.

**M16** ([M+H]^+^, *m/z* 447.0928), **M17** ([M+H]^+^, *m/z* 447.0922), and **M18** ([M+H]^+^, *m/z* 447.0920) were found to have the formula C_21_H_18_O_11_ based on their MS/MS data, which reveals they are 176 Da heavier than **M_0_**. However, the chromatographic retention times were 4.10 min, 4.79 min, and 8.48 min, respectively. Moreover, four dominant ion signals at *m/z* 271.0597 ([M-C_6_H_8_O_6_+H]^+^), 215.0697 ([M-C_6_H_8_O_6_-2CO+H]^+^), 165.0182 (RDA, ^0,3^A), and 153.0181 (RDA, ^1,3^A) suggest that galangin was glucuronidated. The Clog *p*-values of **M16**–**M18** were –0.0529127, 0.444445, and 0.847087, respectively.

**M19** and **M20** afforded [M+H]^+^ ion signals at *m/z* 463.0862 and 463.0859 (C_21_H_18_O_12_) with retention times of 3.41 min and 3.82 min, respectively. They were thought to be the product of an oxidation and glucuronide conjugation reaction and are 16 Da lighter than **M16**–**M18**. Characteristic product ions were observed at *m/z* 329.0508 ([^1,3^A+C_6_H_8_O_6_+H]^+^), 287.0543 ([M-C_6_H_8_O_6_+H]^+^), 165.0184 (^0,3^A), 153.0187 (^1,3^A), and 121.0289 (^0,3^B). The fragment ion signals at *m/z* 329.0508 indicate the possible glucuronidation of the A ring, and the signal at *m/z* 121.0289 indicates that oxidation occurred on the B ring. Because the activity of 4′-H is the strongest on the B ring of flavones, it is likely that 4′-C is oxidized. The molecular formula of both **M19** and **M20** is C_21_H_18_O_12_, and the Clog *p*-values are –0.717049 and 0.182951, respectively.

**M21** had a retention time of 5.68 min and produced a [M+H]^+^ ion signal at *m/z* 351.0169 (C_15_H_10_O_8_S). It is 80 Da heavier than galangin, which means that it may be the product of a sulfation reaction. The presence of MS/MS ion signals at *m/z* 271.0601 ([M-SO_3_+H]^+^), 229.0494 ([M-SO_3_-CO_2_+H]^+^), 165.0183 (^0,3^A), 153.0182 (^1,3^A), and 105.0340 (^0,3^B) confirms this. Therefore, **M21** was identified as the sulfation product of galangin.

**M22** (Clog *p*, −0.301752) and **M23** (Clog *p*, 0.598248), two isomers, gave rise to their theoretical protonated molecular ion signals at *m/z* 367.0115 and 367.0121 (C_15_H_10_O_9_S) at retention times of 5.03 min and 5.44 min, respectively. They were found to be 16 Da heavier than **M21** in positive ion mode, which suggests that they are the products of oxidation and sulfation. They both yielded MS/MS product ions at *m/z* 287.0554, 258.0521, 244.9754 (^0,2^A), 165.0173 ([^0,2^A-SO_3_+H]^+^), 153.0176 ([^1,3^A-SO_3_+H]^+^), and 121.0285 (^0,2^B), which confirms that the oxidation reaction occurs on the B ring and at 4’ on the C ring.

**M24** (*m/z* 285.0758), **M25** (*m/z* 285.0759), and **M26** (*m/z* 285.0755) produced their theoretical deprotonated molecular ions at *m/z* 257.08198 (C_15_H_13_O_4_), and the compounds eluted at 8.01 min, 12.55 min, and 14.82 min, respectively. The methylation reaction could proceed because these compounds are 14 Da heavier than galangin. The spectra of **M24** and **M26** reveal characteristic product ions at *m/z* 270.0513 ([M-CH_3_+H]^+^), 242.0564 ([M-CH_3_-CO+H]^+^), 241.0849 ([M-CO_2_+H]^+^), 211.0744 ([M-CO_2_-OCH_3_+H]^+^), 179.0325 (^0,3^A), 167.0331 (^1,3^A), and 105.0335 (^0,3^B), which demonstrates that the methylation reaction probably occurs on the A ring. The presence of **M25** characteristic product ion signals at *m/z* 270.0533 ([M-CH_3_+H]^+^), 269.0798 ([M-O+H]^+^), 239.0694 ([M-O-OCH_3_+H]^+^), 211.0745 ([M-CO_2_-CH_3_-O+H]^+^), 165.0173 (^0,3^A), 133.0637 (^1,3^B), and 119.0481(^0,3^B) suggests that methylation probably occurred on the B ring. The Clog *p*-values of **M24**–**M26** were 2.50839, 3.00574, and 3.40839, respectively.

**M27**, which is 42 Da heavier than M_0_, eluted at 13.13 min and afforded the deprotonated molecular ion at *m/z* 313.0708 (C_17_H_12_O_6_). Characteristic product ion signals were generated at *m/z* 285.0769 ([M-CO+H]^+^), 257.0798 ([M-2CO+H]^+^), 207.0288 (^0,3^A), 195.0278 (^1,3^A), 119.0491 (^1,3^B), and 105.0337 (^0,3^B) and were tentatively attributed to *N*-acetylation on the A ring.

### 3.5. Proposed Metabolic Pathways of Galangin

In this study, 27 metabolites and 13 metabolic pathways were observed and identified in rats after oral administration of galangin and in vitro. The proposed metabolic pathways of galangin are summarized in Figure 8. Characterization was performed on 27 metabolites in rats (11 in plasma, 15 in bile, 16 in urine, 10 in feces, 3 in heart, 4 in testicles, 4 in muscle, 8 in liver, 8 in bowel, 3 in lung, 15 in kidney, 3 in stomach, 1 in brain, 1 in spleen, and 1 in medullary), 8 metabolites in liver microsomes, and 4 metabolites in intestinal microflora. Structurally, galangin could undergo further biological metabolism, including oxidation, hydrogenation, loss of O, and conjugation reactions (for example, glucuronidation, sulfonation, glucose conjugation, methylation, and acetylation) and their composite reactions, which indicates that galangin is involved in both phase I and II reactions, although oxidation and methylation are the dominant metabolic pathways. However, the N-acetylcysteine conjugation metabolite was only found in urine and feces. To validate the identification and analysis process, we also selected four reference substances (chrysin, apigenin, kaempferol, and kaempferide) and used literature data [20,21,22,23] to verify the rationality of the proposed metabolic pathway.

Galangin metabolites undergo conjugation reactions including glucuronidation, sulfation, acetylation, and methylation. Metabolites that play roles in the oxidation, reduction, and hydrolysis of galangin were detected in plasma samples, urine samples, bile samples, stool samples, and biological tissue samples. Only oxidation, methylation, and glucuronidation reactions were identified in the in vitro metabolism studies. Robert et al. [25] used the Caco-2 cell monolayer model to study the absorption and metabolism of galangin and reported the presence of a galangin glucuronide moiety and sulfate moiety, which is consistent with our results.

### 3.6. Comparison of Metabolites in Different Matrices

Flavonoids do not easily absorb into the bloodstream. Various metabolic enzymes, such as cytochrome P450, UGTs, sulfotransferases, and COMTs, which are highly expressed in the liver and intestinal tract, can promote the absorption and metabolism of flavonoids in vivo. Efflux transporters can also pump metabolites of flavonoids into the intestinal lumen or bile and participate in enterohepatic, intestinal, and local recirculation reabsorption. The combined action of UGTs, efflux transporters, and gut microflora makes these recirculations possible, thereby increasing the residence time of flavonoids in the gut [26] and allowing metabolites to be detected in different matrices such as blood, bile, urine, and feces. In this study, there were more metabolites in the urine and fecal samples than in blood and bile, indicating that urine and feces were the main excretion sites of the active flavonoid metabolites. Flavonoids usually contain multiple hydroxyl groups, which are the key sites for their metabolic transformation in vivo. Galangin was hydroxylated and dehydroxylated in different substrates. It also undergoes methylation, which is a reaction that delays drug clearance and increases drug lipid solubility. Metabolites in feces include glucosylated and methylated products; urine mostly includes phase II metabolites with higher polarities, such as glucuronidation and sulfation metabolites; bile contains not only phase I metabolites, but also phase II glucosylated and methylated metabolites.

### 3.7. Biological Activity of Galangin and Its Metabolites

Once a drug enters the body, it typically undergoes biotransformation through phase I and phase II metabolic pathways. In addition, metabolites resulting from phase I reactions may be chemically reactive and/or pharmacologically active. As shown in FIG. 8, galangin undergoes a phase I reaction to produce various oxidative and reductive metabolites. More and more evidence shows that M11 (chrysin) has strong antitumor activity and inhibits drug resistance through Notch 1, microRNAs, signal transducer and activator of transcription 3 (STAT 3), nuclear factor kappa B (NF-κB), PI 3 K/Akt, MAPK, and other molecular pathways [27]. M1 (apigenin) and M3 (kaempferol) are potential inhibitors of EGFR, HER 2, and MEK 1 [28]. M1 (apigenin) significantly inhibited the activation of MAPK pathways in downstream inflammation by reducing the expression of TNF-α, IL-6, and NF-κB, as well as the expression of apoptotic proteins Bax and caspase-3 [29]. M3 (kaempferol) can also inhibit RhoA/Rho kinase by increasing the release of GLP-1 and insulin, improve renal injury, and reduce fibrosis [30]. Recent studies have shown that galangin-like compounds that lack B ring OH groups may serve as good lead compounds for the rational design of novel COX-2 inhibitors for clinical use as anti-inflammatory drugs by targeting the POX active sites of COX-2 [31]. Therefore, a thorough and comprehensive study of the metabolism of flavonoids in vivo and in vitro can guide the drug screening and structural modification of flavonoids, so as to play a greater role in clinical efficacy.

## 4. Conclusions

A quick and reliable analytical strategy and various post-acquisition data mining tools were successfully implemented to investigate the metabolism of galangin in vitro and in vivo. Although the exact structures of all metabolites could not be unequivocally established through mass spectrometry alone, this study can promote future research on the pharmacology and mechanism of action of galangin. Moreover, many more drug candidates and active compounds with drug potential can be optimized and identified based on the method proposed in this study. More pharmacokinetic data of galangin and its metabolites in vivo are still urgently required for a more comprehensive understanding of the absorption, distribution, and excretion behaviors of different metabolites in organisms.

## Figures and Tables

**Table 1:** Establishment of quality loss template.

No.	Compound Formula	Molecular Weight (Da)	Width (Da)	Mass Loss (mDa)
1	C_15_H_10_O_5_	270.0528	100	52.8236
2	C_15_H_10_O_5_ C_6_H_8_O_6_	446.0849	100	84.9118
3	C_15_H_10_O_5_ SO_3_	350.0096	100	9.6393
4	C_15_H_10_O_5_ CH_2_	284.0685	100	68.4737
5	C_15_H_10_O_5_ C_2_H_2_O	312.0634	100	63.3883

**Table 2:** Summary of phase I and phase II metabolites of galangin in rat liver microsomes, intestinal bacteria, blood, urine, bile, feces, and tissue samples.

Report	Name	Formula	*m/z*	ppm	R.T. (min)	% Score	MS/MS	Clog P	P	B	U	F	H	T	M	Li	Bo	Lu	K	St	Br	S	Me	RML	IB
M1	Isomer	C_15_H_10_O_5_	271.0606	1.8	8.45	94.3	271.0606, 242.0575, 225.0550, 215.0701, 197.0595, 153.0179, 119.0510, 91.0547, 77.0400	2.90529	+	+	+		+	+	+	+	+	+	+		+			+	
M2	Oxidation	C_15_H_10_O_6_	287.0555	1.6	6.47	69.1	287.0555, 259.0604, 258.0527, 231.0658, 203.0719, 165.0203, 153.0201, 135.0458, 121.0287, 93.0336	1.79989	+	+	+			+		+			+						
M3	Oxidation	C_15_H_10_O_6_	287.0547	−1.2	9.39	75.6	287.0547, 259.0607, 258.0523, 231.0655, 203.0717, 165.0193, 153.0194, 135.0455, 121.0288, 93.0334	2.09989			+	+				+								+	+
M4	Oxidation	C_15_H_10_O_6_	287.0556	2.1	10.4	70.8	287.0556, 259.0606, 258.0528, 231.0656, 203.0721, 165.0197, 153.0196, 135.0456, 121.0285, 93.0331	2.09989			+					+		+	+	+					+
M5	Oxidation and Methylation	C_16_H_12_O_6_	301.0711	1.3	8.08	78.1	301.0711, 286.0480, 269.0818, 258.0530, 230.0580, 165.0192, 153.0188, 149.0609, 135.0452, 107.0508	2.13699	+	+	+			+		+	+		+						
M6	Oxidation and Methylation	C_16_H_12_O_6_	301.0715	2.8	8.77	74.9	301.0715, 286.0484, 269.0822, 258.0533, 230.0584, 165.0195, 153.0189, 149.0613, 135.0456, 107.0512	2.69699	+	+			+		+	+	+		+						
M7	Oxidation and Methylation	C_16_H_12_O_6_	301.0712	1.7	12.15	67.7	301.0712, 286.0485, 269.0819, 258.0532, 230.0581, 165.0193, 153.0187, 149.0611, 135.0453, 107.0509	2.69699	+		+	+	+	+	+	+	+	+	+	+		+	+	+	+
M8	Hydrogenation	C_15_H_12_O_5_	273.0762	1.8	6.63	80.8	273.0762, 255.0669, 227.0721, 199.0772, 165.0173, 153.0200, 121.0637, 77.0390	2.03495	+	+	+						+		+						
**Report**	**Name**	**Formula**	***m/z***	**ppm**	**R.T. (min)**	**% Score**	**MS/MS**	**Clog P**	**P**	**B**	**U**	**F**	**H**	**T**	**M**	**Li**	**Bo**	**Lu**	**K**	**St**	**Br**	**S**	**Me**	**RML**	**IB**
M9	Loss of O	C_15_H_10_O_4_	255.0655	1.1	7.74	89.8	255.0655, 227.0711, 211.0768, 199.0761, 181.0657 149.0247, 137.0243, 119.0506, 105.0352	2.49525	+		+	+													
M10	Loss of O	C_15_H_10_O_4_	255.0654	0.7	9.15	76.4	255.0654, 227.0709, 211.0766, 199.0759, 149.0245, 137.0241	3.39525			+	+							+						
M11	Loss of O	C_15_H_10_O_4_	255.0652	0.2	11.73	81.8	255.0652, 209.0585, 153.0179, 103.0549, 77.0392	3.56275			+	+					+							+	
M12	Loss of O and O + Methylation	C_16_H_12_O_3_	253.0865	2.3	6.55	86.4	253.0865, 211.0765, 207.0809, 133.0663, 133.0289, 121.0288, 119.0491, 77.0410	3.089				+													
M13	Loss of O and O + Methylation	C_16_H_12_O_3_	253.0862	1.2	6.90	87	253.0862, 211.0750, 163.0394, 151.0383, 103.0531, 91.0559	3.5865				+							+						
M14	Loss of O and O + Methylation	C_16_H_12_O_3_	253.0861	0.6	8.43	58.8	253.0861, 211.0749, 163.0391, 151.0382, 103.0530, 91.0558	3.5865			+	+							+						
M15 a	Bis-Glucuronide Conjugation	C_27_H_26_O_17_	623.1236	−1.1	4.45	84.4	623.1236, 447.0918, 271.0593	−2.44921		+															
M15 b								−2.08677																	
M15 c								−1.54921																	
M16	Glucuronidation	C_21_H_18_O_11_	447.0928	1.4	4.10	93.5	447.0928, 271.0597, 215.0697, 165.0182, 153.0181	−0.0529127	+	+									+					+	
M17	Glucuronidation	C_21_H_18_O_11_	447.0922	0	4.79	88.9	447.0922, 271.0595, 215.0694, 165.0180, 153.0183	0.444445	+	+	+								+					+	
M18	Glucuronidation	C_21_H_18_O_11_	447.0920	−0.5	8.48	75	447.0920, 271.0593, 215.0691, 165.0177, 153.0179	0.847087	+	+									+					+	
M19	Oxidation and Glucuronide Conjugation	C_21_H_18_O_12_	463.0862	−1.8	3.41	75.6	463.0862, 329.0508, 287.0543, 165.0184, 153.0187, 121.0289	−0.717049		+															
M20	Oxidation and Glucuronide Conjugation	C_21_H_18_O_12_	463.0859	−2.6	3.82	74.1	463.0859, 329.0506, 287.0540, 165.0181, 153.0184, 121.0285	0.182951		+															
**Report**	**Name**	**Formula**	***m/z***	**ppm**	**R.T. (min)**	**% Score**	**MS/MS**	**Clog P**	**P**	**B**	**U**	**F**	**H**	**T**	**M**	**Li**	**Bo**	**Lu**	**K**	**St**	**Br**	**S**	**Me**	**RML**	**IB**
M21 a	Sulfate Conjugation	C_15_H_10_O_8_S	351.0169	−0.2	5.68	96.4	351.0169, 271.0601, 229.0493, 165.0183, 153.0182, 105.0340	0.362386			+														
M21 b								1.26239																	
M21 c								1.41974																	
M22	Oxidation and Sulfate Conjugation	C_15_H_10_O_9_S	367.0115	−0.8	5.03	79.6	367.0115, 287.0554, 258.0521, 244.9754, 165.0173, 153.0176, 121.0285	−0.301752		+															
M23	Oxidation and Sulfate Conjugation	C_15_H_10_O_9_S	367.0121	0.8	5.44	82.1	367.0121, 287.0558, 258.0526, 244.9759, 165.0177, 153.0179, 121.0287	0.598248		+															
M24	Methylation	C_16_H_12_O_5_	285.0758	0.3	8.01	74.4	285.0758, 270.0513, 242.0564, 241.0849, 211.0744, 179.0325, 167.0331, 105.0335	2.50839	+	+	+					+	+		+						
M25	Methylation	C_16_H_12_O_5_	285.0759	0.5	12.55	96.3	285.0759, 270.0533, 269.0798, 239.0694, 211.0745, 165.0173, 133.0637, 119.0481	3.00574			+	+			+		+		+	+				+	
M26	Methylation	C_16_H_12_O_5_	285.0755	−0.9	14.82	97.1	285.0755, 270.0511, 242.0561, 241.0845, 211.0742, 179.0322, 167.0328, 105.0331	3.40839			+														+
M27 a	N-Acetylation	C_17_H_12_O_6_	313.0708	0.6	13.13	84.9	313.0708, 285.0769, 257.0798, 207.0288, 195.0278, 119.0491, 105.0337	1.93839			+	+													

Note: +: Detected. a, b and c: possible metabolites. P: plasma, B: bile, U: urine, F: feces. H: heart, T: testicles, M: muscle, Li: liver, Bo: bowel, Lu: lung, K: kidney, St: stomach, Br: brain, S: spleen, Me: medullary. RML: rat liver microsome. IB: intestinal bacteria.

## Data Availability

The data presented in this study are available in the main article.
